# Supplementary material for: Beyond volume and toward coherence: a research parasite’s perspective
Source: Gigascience. 2026 Jan 20;15:giag001. doi: 10.1093/gigascience/giag001 (PMC12927410; doi:10.1093/gigascience/giag001)
Supplement: giag001_GIGA-D-25-00521_Revision_1 [file giag001_giga-d-25-00521_revision_1.pdf]

GigaScience

# Beyond Volume and Toward Coherence: A research parasite's perspective

--Manuscript Draft--

| | |
|---|---|
| **Manuscript Number:** | GIGA-D-25-00521R1 |
| **Full Title:** | Beyond Volume and Toward Coherence: A research parasite's perspective |
| **Article Type:** | Commentary |
| **Funding Information:** | |
| **Abstract:** | The Pacific Symposium on Biocomputing (PSB) recognized my work with the 2024 Junior Research Parasite Award, an honor established to highlight the scientific value of reanalyzing, integrating, and reinterpreting existing datasets. The award invites recipients to reflect on the role of research parasites within the broader ecosystem of computational biology and data reuse. For me, this perspective is rooted in years of working across diverse -omics datasets, where I've seen firsthand how the structure, resolution, and context of a dataset shape the biological insight it can support. Rather than focusing on data volume alone, meaningful discovery often emerges from understanding what each dataset can—and cannot—reveal. Here, I outline how different modes of secondary analysis, from integrating complementary datasets to deeply mining a single omics layer. |
| **Corresponding Author:** | Gina Turco<br>Golgi Inc<br>San Francisco, CA UNITED STATES |
| **Corresponding Author Secondary Information:** | |
| **Corresponding Author's Institution:** | Golgi Inc |
| **Corresponding Author's Secondary Institution:** | |
| **First Author:** | Gina Turco |
| **First Author Secondary Information:** | |
| **Order of Authors:** | Gina Turco |
| **Order of Authors Secondary Information:** | |
| **Response to Reviewers:** | Thank you very much for the prompt review of my commentary. I've revised the submission accordingly.<br><br>Background/Introduction<br><br>I have added a separate, clearly labeled Background/Introduction section to align with the required Commentary structure.<br><br>Data Availability and List of Abbreviations<br><br>I have included a Data Availability statement marked as "Not applicable" and added a complete List of Abbreviations.<br><br>Reference Count<br><br>I have reduced the reference list from 12 to 10 by removing two non-essential citations.<br><br>The revised manuscript has been uploaded through the online submission system. Thank you again for your help and feedback. I appreciate the opportunity to submit this perspective. |
| **Additional Information:** | |
| **Question** | **Response** |

*Powered by Editorial Manager® and ProduXion Manager® from Aries Systems Corporation*

| | |
|---|---|
| Are you submitting this manuscript to a special series or article collection? | No |
| **Experimental design and statistics**<br><br>Full details of the experimental design and statistical methods used should be given in the Methods section, as detailed in our Minimum Standards Reporting Checklist. Information essential to interpreting the data presented should be made available in the figure legends.<br><br>Have you included all the information requested in your manuscript? | No |
| If not, please give reasons for any omissions below.<br><br>as follow-up to "**Experimental design and statistics**<br><br>Full details of the experimental design and statistical methods used should be given in the Methods section, as detailed in our Minimum Standards Reporting Checklist. Information essential to interpreting the data presented should be made available in the figure legends.<br><br>Have you included all the information requested in your manuscript?<br><br>" | The full experimental design and stats are reported in cited papers of the perspective |
| **Resources**<br><br>A description of all resources used, including antibodies, cell lines, animals and software tools, with enough information to allow them to be uniquely identified, should be included in the Methods section. Authors are strongly encouraged to cite Research Resource Identifiers (RRIDs) for antibodies, model organisms and tools, where possible. | No |

| | |
|---|---|
| Have you included the information requested as detailed in our Minimum Standards Reporting Checklist? | |
| If not, please give reasons for any omissions below.<br><br>as follow-up to "**Resources**<br><br>A description of all resources used, including antibodies, cell lines, animals and software tools, with enough information to allow them to be uniquely identified, should be included in the Methods section. Authors are strongly encouraged to cite Research Resource Identifiers (RRIDs) for antibodies, model organisms and tools, where possible.<br><br>Have you included the information requested as detailed in our Minimum Standards Reporting Checklist?<br><br>" | The full resources are reported in cited papers of the perspective |
| **Availability of data and materials**<br><br>All datasets and code on which the conclusions of the paper rely must be either included in your submission or deposited in publicly available repositories (where available and ethically appropriate), referencing such data using a unique identifier in the references and in the "Availability of Data and Materials" section of your manuscript.<br><br>Have you have met the above requirement as detailed in our Minimum Standards Reporting Checklist? | No |
| If not, please give reasons for any omissions below.<br><br>as follow-up to "**Availability of data and** | The data availability are reported in cited papers of the perspective |

| | |
|---|---|
| **materials**<br><br>All datasets and code on which the conclusions of the paper rely must be either included in your submission or deposited in publicly available repositories (where available and ethically appropriate), referencing such data using a unique identifier in the references and in the "Availability of Data and Materials" section of your manuscript.<br><br>Have you have met the above requirement as detailed in our Minimum Standards Reporting Checklist?<br><br>" | |
| GigaScience has policies and guidelines in place for the use of generative AI-writing tools such as ChatGPT. If you have used such writing tools to assist with writing the manuscript this must be declared and cited in the text. Authors should not list AI-writing tools and other AI-assisted technologies as an author or co-author and should acknowledge that they are fully responsible for text generated or refined by AI-writing tools.<p><br><br>A summary of use (particularly in the introduction or among methods) needs to be included at the end of the paper, and the outputs should also be included as a supplementary file hosted in GigaDB or other open repositories. Please <a href=https://academic.oup.com/gigascience/pages/editorial_policies_and_reporting_standards target="_new" > read our guidelines for more information. </a> <p><br><br>By submitting to GigaScience, you are aware of the journal's AI-writing tools policy, and if you have declared use of such tools below, you have acknowledged this where appropriate in your manuscript and have made a summary of use and outputs available. </b><p><br><b>AI-assisted writing tools have been used in the preparation of this manuscript? | No |

Manuscript
Click here to access/download;Manuscript;Turco_Data_Parasite_revisions

# Beyond Volume and Toward Coherence: A research parasite's perspective

## Abstract

The Pacific Symposium on Biocomputing (PSB) recognized my work with the **2024 Junior Research Parasite Award**, an honor established to highlight the scientific value of reanalyzing, integrating, and reinterpreting existing datasets. The award invites recipients to reflect on the role of research parasites within the broader ecosystem of computational biology and data reuse. For me, this perspective is rooted in years of working across diverse -omics datasets, where I've seen firsthand how the structure, resolution, and context of a dataset shape the biological insight it can support. Rather than focusing on data volume alone, meaningful discovery often emerges from understanding what each dataset can—and cannot—reveal. Here, I outline how different modes of secondary analysis, from integrating complementary datasets to deeply mining a single omics layer.

## Background

We live in a data-rich era, often with more -omics data than we know what to do with. When working with diverse -omics data types, I've found that the type of data strongly shapes the emergent properties we can uncover. Different datasets carry distinct strengths tied to the biological questions being asked, whether using complementary -omics datasets to identify mechanisms of action, combining similar datasets to detect subtle patterns that no single experiment could reveal, or performing discovery within a dataset by leveraging metadata to infer structure. As the recipient of the **2024 Junior Research Parasite Award**, I was invited to share my perspective on generating new hypotheses from existing data, and I felt the most meaningful way to do so was through examples drawn from my own work.

It has always been a dream of mine to model the cell. Specifically, I want to be able to characterize the promoter regions that are functional under a given condition. The readout of this system signals what genes are transcribed and what proteins are translated thus leading to coordinated changes occurring in the organism. Even with extensive -omics data, this remains difficult. Biology is complex and no single experiment can address all the complexities of noisy and hard to control environments. This makes it challenging to decode mechanisms of action within a specific context, such as a particular cell type or stress condition. I have found that there are two types of data analysis: inter- and intra- dataset discovery that help disentangle some of these complexities. In **intra-dataset discovery**, we focus on extracting as much insight as possible from a single dataset, looking for patterns, trends, and mechanistic signals within the same -omics layer. In **inter-dataset discovery**, multiple complementary -omics datasets are

integrated to tell a more complete and cohesive biological story. Both types of data discovery require a deep understanding of the biology behind the data, including experimental design and technical methods used to generate the data, the questions being asked, and how the data can help answer those questions. The most effective models employ highly specific, high-resolution -omics datasets tailored to the precise questions being asked. This specialized data serves as a valuable prior for learning from the current data.

## Inter-dataset discovery: using multiple -omics datasets to complement one another

The key to inter-dataset discovery is having a clear understanding of the scientific questions you are trying to answer, the strengths and limitations of each dataset collected to answer those questions, and the potential caveats inherent to each data type. Inter-dataset discovery arises when different yet complementary -omics layers are combined to deepen biological insight.

One example from my own work of inter-dataset discovery comes from studying gene regulatory networks (GRNs) involved in drought tolerance in plants [1]. Because water transport is tightly linked to drought response, I focused on the tissue most directly responsible for this process: the xylem. To build a tissue-specific network, we performed yeast one-hybrid (Y1H) assays using only transcription factors expressed in xylem cells to look for transcription factor-promoter interactions. Even with this cell-type–specific restriction, the resulting network remained large and highly complex, consisting of 621 transcription factor-promoter interactions [1]. This level of complexity is common in GRNs, which often expand into structures that are so dense and interconnected they are difficult to interpret and frequently referred to as “hairballs”.

The key to disentangling the network and understanding which connections were meaningful involved incorporating complementary datasets. A key limitation of Y1H assays is that transcription factor expression depends on environmental context (e.g., whether the plant is under stress). Similarly, promoter regions are not guaranteed to be accessible in all conditions. It was therefore essential to complement our Y1H data with xylem-specific expression time-course data that captured gene expression changes under multiple drought stress conditions [1]. In addition to gene expression data, we integrated xylem-specific bisulfite sequencing, which provided information on promoter accessibility and DNA methylation status [2]. Together, these complementary datasets enabled us to refine the network and identify key regulatory hubs.

Another example of integrating complementary datasets came from single-cell RNA-seq analysis of the *Arabidopsis thaliana* root. Generating this data was difficult because this was the first single-cell transcriptomics successfully applied to plants [3]. The presence of a rigid cell wall and large, non-uniform size of plant cells made cell isolation technically challenging. Even after successful isolation, single-cell RNA-seq data tended to be sparse and noisy, making it difficult

to assign clear cell identities. To address this, I combined two complementary data types: single-cell RNA-seq data, which captures fine-grained variation across individual cells but is inherently noisy, and high-resolution tissue-specific microarray data from FACS-sorted root cells, which provides stable, averaged expression profiles for known cell types [4]. This prior information—on which genes have high, low, or mid expression (also specificity broadly or narrowly expressed across cell types) in which cell types—allowed us to infer how much information a gene's expression levels contributed to cell identity and how much weight should be given to each gene for a given cell type [3,5]. This also allowed us to give individual cells from single-cell analysis a cell identity score independent of the single-cell's t-SNE clustering. Using both techniques, we were able to confidently define cell types in the single-cell data and better identify mixed or still-differentiating cell types, including cell types like the xylem that undergo programmed cell death upon differentiation [5].

## New discoveries via intra-dataset discovery

I**ntra-dataset discovery** is defined as a deep understanding for the -omics data type you already have the ability to identify patterns and make inferences from them. These are often cases where the same pattern is repeated across experiments, across separate labs and various conditions, and thus likely reflective of underlying truth. This type of discovery is ideal for building inference models that can add confidence and statistical power to analysis. Intra-dataset discovery requires a deep understanding of the data and how it was generated, and is therefore best achieved through close collaboration between experimentalists and analysts. Such understanding of experimental and analytical complexity allows analysts to directly address data artifacts and generate insights that would otherwise be missed.

At my current company, Golgi Inc, we are fortunate to work closely with the scientists generating the proteomics data that we analyze. We can therefore use the experimental knowledge to better leverage the metadata generated in these proteomics experiments with our models to produce more accurate results. For example, intensity values within proteomics datasets are heterogeneous; some measurements are of high quality, while others are less reliable. Generally, the quality of the measurements is largely determined by the number of ions captured in the Orbitrap. Understanding the relationship between technical variables and measurement quality—for example, that higher ion counts generally correspond to more reliable measurements—makes it possible to uncover patterns that would otherwise be consumed by technical noise. Through variance modeling, heteroskedastic models [6], and variance moderation techniques [7], the re-analysis looks more like a fundamental improvement in the underlying technology. Opportunities to improve data insight only grow with technological complexity.  For example, in Data Independent Acquisition (DIA) the relationship between ion count and quality becomes more complicated due to interference from overlapping fragment ions. By integrating metadata—such as spectral quality and identification scores, we can

generate robust weights that overcome these interferences and improve proteomic results. Benchmarking our weighted strategy against standard methods (such as MaxLFQ [8]) shows higher precision, particularly for mid-low range peptides. In controlled DIA experiments with known ground truth (2-fold change), our weighted approach reduces variance by up to 41%, bringing protein estimates much closer to the expected 2-fold change. This is one of many examples where the ground-truth is hidden in noise but by understanding the experiment, instruments used, and sources of error we can leverage that knowledge to integrate the noise into our models so they better reflect the true biology.

This familiarity with how data are produced makes it easier to build systems that are robust to error and grounded in experimental reality. Conversely, analyzing datasets generated by external groups, without direct interaction with the experimentalists, introduces additional uncertainty and complexity. Metadata may be sparse, protocols vary, and batch effects can be difficult to disentangle. Still, with enough data, new patterns can emerge, especially when results replicate across multiple experiments, platforms, and research groups. Convergent evidence is almost always more reflective of underlying biological truth than any single dataset.

My work on the Yeast Phenome is an example of this [9]. The release of the yeast knockout (YKO) collection in 2002 enabled comprehensive assays of gene function across nearly every measurable aspect of yeast biology [10]. While hundreds of labs produced valuable loss-of-function screens, the results remained scattered and inconsistently annotated, limiting any ability to integrate them. Yeast Phenome was built to solve this problem by aggregating and harmonizing all published screens of the YKO collection. The resource currently contains ~43 million gene-to-phenotype links extracted from 531 papers across 366 laboratories—the largest and most systematic phenotypic description for any organism [9]. Conceptually, it functions as a massive data matrix: each row is a knockout strain, each column is a phenotypic screen, and each entry is an annotated measurement linked to standardized vocabularies describing the phenotype and the experimental conditions under which it was tested.

By combining all screens into a unified structure, we could detect patterns that would have been invisible within any individual dataset. One example is the relationship between phenotypic similarity and intergenic distance. Because each gene had multiple phenotype–experiment measurements, we could compute pairwise phenotypic correlations across the entire chromosome. When viewed in aggregate, an exponential increase in phenotypic similarity with chromosomal proximity emerged [9], an insight impossible to detect without comprehensive data integration.

## Conclusions

Understanding biological data requires more than algorithms—it takes combined expertise between biologists and data scientists who understand where the data came from and what

questions are being asked. With increasing amounts of -omics data and the capability for artificial intelligence to use all the data we may naively expect these models to perform better, but success does not only depend on quantity. Quality, context, and reproducibility matter just as much.

Effective analytical approaches ask: Do the data truly answer the biological question being asked? What relationships can be leveraged with the current data? How consistently do results replicate across experiments and research groups? While no single experiment can tell the whole story, biological mechanisms start to emerge when diverse datasets are integrated thoughtfully, guided by biological insight and grounded in reproducible evidence.

## Note from the Editors

The Research Parasite Awards take place at the Pacific Symposium on Biocomputing each January at the Fairmont Orchid on the Big Island of Hawaii, USA. The establishment of the award was a reaction to an editorial that presented arguments against data sharing, including that it promoted a system where "research parasites" (those who reuse datasets created by "frontline researchers") would proliferate. As promoters of data sharing, GigaScience Press has supported the Junior Parasite Award for postdoctoral, graduate, or undergraduate trainees. Publishing Commentaries from the winners provides useful lessons for other research parasites. For more, see the Research Parasite Awards website, https://researchparasite.com/.

## Abbreviations

RNA-seq: RNA sequencing; scRNA-seq: single-cell RNA sequencing; TF: transcription factor; t-SNE: t-distributed stochastic neighbor embedding; Y1H: yeast one-hybrid; YKO: yeast knockout collection; DIA: Data-Independent Acquisition; FACS: fluorescence-activated cell sorting; GRN: gene regulatory network; LFQ: label-free quantification; MaxLFQ: maximal peptide ratio extraction algorithm for LFQ quantification; MS: mass spectrometry; QC: quality control.

## Data Availability

Not applicable.

## Competing Interests

All authors are employees of Golgi Inc.

# Citations

[1] Taylor-Teeples, M., Lin, L., de Lucas, M., Turco, G., Toal, T. W., Gaudinier, A., et al. (2014). An Arabidopsis gene regulatory network for secondary cell wall synthesis. Nature, 517(7536), 571–575. https://doi.org/10.1038/nature14099

[2] Turco, G. M., Kajala, K., Kunde-Ramamoorthy, G., Ngan, C.-Y., Olson, A., Deshpande, S., et al. (2017). DNA methylation and gene expression regulation associated with vascularization in Sorghum bicolor. New Phytologist, 214(3), 1213–1229. https://doi.org/10.1111/nph.14533

[3] Shulse, C. N., Cole, B. J., Ciobanu, D., Lin, J., Yoshinaga, Y., Gouran, M., et al. (2019). High-throughput single-cell transcriptome profiling of plant cell types. Cell Reports, 27(7), 2241–2247.e4. https://doi.org/10.1016/j.celrep.2019.04.054

[4] Birnbaum, K., Jung, J. W., Wang, J. Y., Lambert, G. M., Hirst, J. A., Galbraith, D. W., et al. (2005). Cell type–specific expression profiling in plants via cell sorting of protoplasts from fluorescent reporter lines. Nature Methods, 2(8), 615–619. https://doi.org/10.1038/nmeth0815-615

[5] Turco, G. M., Rodriguez-Medina, J., Siebert, S., Han, D., Valderrama-Gómez, M. Á., Vahldick, H., et al. (2019). Molecular mechanisms driving switch behavior in xylem cell differentiation. Cell Reports, 28(2), 342–351.e4. https://doi.org/10.1016/j.celrep.2019.06.047

[6] O'Brien, J. J., Raj, A., Gaun, A., Waite, A., Li, W., Hendrickson, D. G., et al. (2024). A data analysis framework for combining multiple batches increases the power of isobaric proteomics experiments. Nature Methods, 21(2), 290–300. https://doi.org/10.1038/s41592-023-02065-4

[7] Law, C. W., Chen, Y., Shi, W., & Smyth, G. K. (2014). voom: Precision weights unlock linear model analysis tools for RNA-seq read counts. Genome Biology, 15(2), R29. https://doi.org/10.1186/gb-2014-15-2-r29

[8] Cox, J., Hein, M. Y., Luber, C. A., Paron, I., Nagaraj, N., & Mann, M. (2014). Accurate proteome-wide label-free quantification by delayed normalization and maximal peptide ratio extraction, termed MaxLFQ. Molecular & Cellular Proteomics, 13(9), 2513–2526. https://doi.org/10.1074/mcp.M113.031591

[9] Turco, G., Chang, C., Wang, R. Y., Kim, G., Stoops, E. H., Richardson, B., et al. (2023). Global analysis of the yeast knockout phenome. Science Advances, 9(21), eadg5702. https://doi.org/10.1126/sciadv.adg5702

[10] Giaever, G., Chu, A. M., Ni, L., Connelly, C., Riles, L., Véronneau, S., et al. (2002). Functional profiling of the Saccharomyces cerevisiae genome. Nature, 418, 387–391. https://doi.org/10.1038/nature00935
